# Mast cells promote pathology and susceptibility in tuberculosis

**DOI:** 10.1101/2024.09.04.611333

**Published:** 2024-09-09

**Authors:** Ananya Gupta, Vibha Taneja, Javier Rangel Moreno, Abhimanyu, Mushtaq Ahmed, Nilofer Naqvi, Kuldeep S. Chauhan, Daniela Trejo-Ponce de León, Gustavo Ramírez-Martínez, Luis Jiménez-Alvarez, Cesar Luna-Rivero, Joaquin Zuniga, Deepak Kaushal, Shabaana A. Khader

**Affiliations:** 1 The University of Chicago, Department of Microbiology, 920 East 58^th^ Street, CLSC 1117, Chicago, IL 60637.; 2 Department of Molecular Microbiology, Washington University in St. Louis, St. Louis, MO 63110, USA; 3 Division of Allergy, Immunology and Rheumatology, Department of Medicine, University of Rochester Medical Center, Rochester, NY, USA; 5 Technologico de Monterrey, Escuela de Medicina y Ciencias de la Salud, Mexico City, Mexico; 6 Laboratory of Immunobiology and Genetics and Department of Pathology, Instituto Nacional de Enfermedades Respiratorias Ismael Cosio Villegas, Mexico; 7 Texas Biomedical Research Institute, San Antonio, Texas 78245-0549, USA

**Keywords:** Mast cells, Tuberculosis, lung, innate cell, inflammation

## Abstract

Tuberculosis (TB), caused by the bacterium *Mycobacterium tuberculosis* (*Mtb*), infects approximately one-fourth of the world’s population. While most infected individuals are asymptomatic, latent TB infection (LTBI) can progress to cause pulmonary TB (PTB). We recently reported an increased accumulation of mast cells (MCs) in lungs of macaques with PTB, compared with LTBI in macaques. MCs respond in vitro to *Mtb* exposure via degranulation and by inducing proinflammatory cytokines. In the current study, we show the dominant production of chymase by MCs in granulomas of humans and macaques with PTB. Using scRNA seq analysis, we show that MCs found in LTBI and healthy lungs in macaques are enriched in genes involved in tumor necrosis factor alpha, cholesterol and transforming growth factor beta signaling. In contrast, MCs clusters found in PTB express transcriptional signatures associated with interferon gamma, oxidative phosphorylation, and MYC signaling. Additionally, MC deficiency in the mouse model showed improved control of *Mtb* infection that coincided with reduced accumulation of lung myeloid cells and diminished inflammation at chronic stages. Thus, these collective results provide novel evidence for the pathological contribution of MCs during *Mtb* infection and may represent a novel target for host directive therapy for TB.

## Introduction.

Tuberculosis (TB) remains a significant global health issue, with approximately one-quarter of the world’s population harboring *Mycobacterium tuberculosis* (*Mtb*) causing around 1.5 million deaths each year (WHO, 2023). The disease often starts as a latent TB infection (LTBI), in which the bacteria remain dormant and cause no symptoms. However, LTBI can progress to active pulmonary TB (PTB), characterized by severe respiratory symptoms and high transmission potential. The immune mechanisms that allow progression from latent to PTB are not fully defined. Thus, understanding the immune factors that drive progression toward PTB will allow the development of novel therapeutics for TB control. Towards this overall goal, we recently showed that the lung single-cell transcriptional immune landscape during LTBI and PTB in macaques infected with *Mtb* was distinct. For example, PTB was characterized by the significant accumulation of Type I IFN expressing-plasmacytoid DCs (pDCs), IFN-responsive macrophages as well as activated T cells to the lungs, (Esaulova et al., 2021). Additionally, mast cells (MCs) were increased in the lungs of macaques with PTB (Esaulova et al., 2021). In sharp contrast, LTBI was characterized by increased presence of cytotoxic NK cells but lack of recruitment of MCs in the lungs (Esaulova et al., 2021).

MCs are found in the lung where they influence inflammatory responses (Naqvi et al., 2021). MCs have been shown to respond *in vitro* to *Mtb* exposure via surface receptors such as CD48 (Munoz et al., 2003). They also react to *Mtb* exposure or mycobacterial lipids by undergoing degranulation of prestored granules such as histamine and β-hexosaminidase, and inducing secretion of proinflammatory cytokines such as IL-6 and TNF-α (Munoz et al., 2003). Degranulation of MCs following intratracheal infection with a high dose of *Mtb* resulted in limiting inflammation and proinflammatory cytokines such as IL-1β and TNF-α (Carlos et al., 2007). MCs produce and release either chymase or tryptase (Bian et al., 2021) which are both proteases that are stored in the cell’s secretory granules. Recent studies with lung biopsies of TB patients showed an enrichment of MCs expressing IL-17 at inflammatory sites. In contrast, chymase rich MCs (MC_C_s) producing TGF-β were detected in proximity to mature granulomas in lung biopsies from PTB (Garcia-Rodriguez et al., 2021). Furthermore, while healthy lung predominantly has tryptase-expressing mast cells (MC_T_s), both chymase and dual mast cells co-expressing chymase and tryptase (MC_C_s and MC_TC_s) accumulate in the infected lung of patients with PTB (Garcia-Rodriguez et al., 2021). Thus, while previous studies have shown that MCs respond to *Mtb* exposure and accumulate in macaque and human lungs during PTB, it is not completely known if MCs functionally mediate protective or pathological outcomes in the context of TB infection.

In the current study, we show that the distribution and localization of MCs in PTB in humans and macaques is associated with chymase production. Using scRNA seq analysis, we show that MCs found in LTBI and healthy lungs in macaques, are transcriptionally distinct than PTB lungs showing enrichment of tumor necrosis factor alpha, cholesterol and transforming growth factor beta signaling, while MCs found in PTB express increased levels of signatures associated with interferon gamma, oxidative phosphorylation, and MYC signaling. Additionally, mice deficient in MCs, showed improved control of *Mtb* infection and reduced lung inflammation, providing novel evidence that chymase positive MCs contribute to immune pathology and reduced *Mtb* control, suggesting a pathological role for MCs during *Mtb* infection.

## Materials and methods

### Study subjects

All human lung biopsy samples were obtained from the Tuberculosis Outpatient Clinic and the Department of Pathology at the National Institute of Respiratory Diseases (INER) in Mexico City, before *M. tuberculosis* treatment with informed consent, and with the approved protocol by the INER IRB for their use (project numbers B04–15 and B09–23). Also, lung samples from healthy controls (HC) non-TB individuals were obtained from the tissue repository of the Department of Pathology from INER. No compensation was provided to the patients.

Non-human primate procedures were approved by the Institutional Animal Care and Use Committee of Tulane National Primate Research Center and were performed in accordance with National Institutes of Health (NIH) guidelines. Male and female Indian rhesus macaques, verified to be free of *Mtb* infection by tuberculin skin test, were obtained from the Tulane National Primate Research Center. The animals were housed in an ABSL3 facility.

C57Bl/6 and B6.Cg-KitW-sh/HNihrJaeBsmJ (Strain #:030764) alias *CgKitW*^*sh*^ mice were procured from Jackson Laboratory (Bar Harbor, ME) and bred at Washington University in St. Louis. Six to eight weeks female and male mice were used in the experiments. All mice were maintained and used in accordance with the approved Institutional Animal Care and Use Committee (IACUC) guidelines at Washington University in St. Louis.

### Aerosol Infection

*Mtb* strain HN878 was cultured in Proskauer Beck medium containing 0.05% Tween 80 until reaching mid-log phase and frozen in 1 ml aliquots at −80°C until used. Mice were aerosol infected with ~100 colony forming units (CFU), as described previously (Khader et al., 2007). Macaques were assigned to three groups: (1) uninfected control, (2) macaques with LTBI were exposed to a low dose (~10 CFU), and (3) macaques with PTB were exposed to a high dose (~100 CFU) of *Mtb* CDC1551 via the aerosol route using a custom head-only dynamic inhalation system housed within a class III biological safety cabinet as previously described (Esaulova et al., 2021). The animals were periodically monitored for detecting alterations in their physiological parameters and to monitor disease symptoms.

### Bacterial burden and cytokine Analysis

Bacterial burden was assessed using serial 10-fold dilutions of lung or spleen homogenates and plated on 7H11 agar solid medium supplemented with OADC (oleic acid, bovine albumin, dextrose, and catalase). Colonies were counted after 2–3 weeks of incubation.

Cytokine/chemokine expression was analyzed in lung homogenates from infected mice via Luminex (Millipore-Sigma) or ELISA (R&D) as per the manufacturer’s protocol.

### Generation of single-cell suspensions from tissues and flow cytometry staining

Lung single-cell suspensions from *Mtb*-infected mice were prepared as previously described (Gopal et al., 2013). Briefly, mice were euthanized with CO_2_. The right lower lobe was isolated and perfused with heparin in saline. Lungs were minced and incubated with collagenase/DNAse for 30 minutes at 37°C. Lung tissue was pushed through a 70μm nylon screen to obtain a single cell suspension. Following lysis of erythrocytes, the cells were washed and resuspended in cDMEM (DMEM high glucose + 10% fetal bovine serum + 1% Penicillin/Streptomycin) for flow cytometry staining. For flow cytometric analysis, cells were either stained immediately, or stimulated with phorbol myristate acetate (PMA-50ng/ml; Sigma Aldrich) and ionomycin (750 ng/ml; Sigma Aldrich) in the presence of Golgistop (BD Pharmingen).

The following fluorochrome conjugated antibodies were used for myeloid cell surface staining CD11b APC (clone M1 / 70), CD11c PeCy7 (clone HL3, BD Biosciences) GR-1 PerCP-Cy5.5 (Clone RB6–8C5, BD Pharmingen) and MHC class II FITC (Clone M5/114.15.2, Tonbo Biosciences), Anti-Mo CD117 (cKit) super bright 780 (eBiosciences; clone 2BB), FcεR1 PE (eBiosciences; clone MAR-1). Myeloid cells: alveolar macrophages were gated on CD11c^+^CD11b^-^, neutrophils were defined as CD11b^+^CD11c^-^Gr-1^hi^ cells, monocytes were defined as CD11b^+^CD11c^-^Gr-1^med^ cells, recruited macrophages were defined as CD11b^+^CD11c^-^Gr-1^low^ cells and mast cells were defined as CD11b^-^cKit^+^FcεR1^+^ cells. T cells were identified based on gating strategy as described before (Griffiths et al., 2016). CD3 AF700 (clone 500A2, BD Biosciences), CD4 Pacific blue (clone RM4.5, BD Biosciences), CD44 PeCy7 (clone 1M7, Tonbo Biosciences), CD8 APC Cy7 (clone 53–6.7, BD Biosciences) were used for T-cell surface staining. Fixation/permeabilization concentrate and diluent (eBioscience) were used in the intracellular stain to fix and permeabilize lung cells following manufacturer’s instructions. Intracellular staining was performed with IFNγ APC (clone XMG1.2, Tonbo Biosciences) and TNF-α -FITC (Clone MP6-XT22, BD Pharmingen) or the respective isotype control antibodies (APC rat IgG1κ and FITC rat IgG1α isotype, BD Pharmingen) for 30 min. Samples were acquired on a 4 laser BD Fortessa Flow Cytometer and the analysis was performed using FlowJo (Treestar). Total numbers of cells within each gate were back calculated based on cell counts in the individual lung samples.

### Histological analysis

For mice studies, the left upper lobe was collected for histomorphometric analysis. The lobes were infused with 10% neutral buffered formalin and embedded in paraffin. 5 μm thick ling sections were cut using a microtome, stained with hematoxylin and eosin (H&E) and processed for light microscopy. Images were captured using the automated Nanozoomer digital whole slide imaging system (Hamamatsu Photonics). Regions of inflammatory cell infiltration were delineated utilizing the NDP view2 software (Hamamatsu Photonics), and the percentage of inflammation was calculated by dividing the inflammatory area by the total area of individual lung lobes. All scoring was conducted in a blinded manner.

FFPE lung sections from healthy individuals, tuberculosis patients and NHP infected with *MTB* were stained with goat anti-human mast cell chymase (LifeSpan Biosciences, LS-B4134, RRID:AB_10718418) and rabbit anti-human tryptase (Cell Signaling Technology, 195235). Primary antibodies were detected with Alexa fluor 568 donkey anti-goat Ig G (Thermo Fisher Scientific, A-11057, RRID:AB_2534104) and Alexa Fluor 488 donkey anti-rabbit Ig G (Jackson ImmunoResearch Laboratories, 711–546-152, RRID:AB_2340619). Nuclei were labeled with DAPI. MC positive for chymase, tryptase or both were blindly quantitated in 3 200x random fields per sample in human and NHP lung sections. 200x pictures were taken with an Axioplan Zeiss microscope and recorded with a Hamamatsu camera.

### Data analysis and statistics

All data was analyzed using the indicated methodology in each figure legend. Two sided-unpaired t-test was performed for comparing significance between 2 groups, one-way ANOVA Tukey’s test and two-way ANOVA Sidak’s multiple comparison test were performed for more than 2 groups using GraphPad Prism 5 and 10, respectively (La Jolla, CA). Significance is denoted on the figure and the respective figure legends. Outliers, if any, were removed using Grubb’s outlier test and mentioned in the respective figures.

### Single cell data reanalysis

The NHP single cell lung data was accessed from GEO (GSE149758) and processed through *cellranger 7.0* using the Macaca Mulatta reference genome (Mmul_10). The obtained matrix file was processed through the R package *Seuratv- 5* for downstream analysis of the count matrix. The cells were filtered based on mitochondrial gene content and at least 500 genes detected. Data was log normalized. The most variable genes were detected by the *FindVariableFeatures* function and used for subsequent analysis. Latent variables (number of UMI’s and mitochondrial content) were regressed out using a negative binomial model (function *ScaleData*). Principle component analysis (PCA) was performed with the *RunPCA* function. A UMAP dimensionality reduction was performed on the scaled matrix (with most variable genes only) using the first 20 PCA components to obtain a two-dimensional representation of the cell states. For clustering, we used the functions *FindNeighbors* (20 PCA) and *FindClusters* (resolution 0.25). MCs were identified and re-clustered based on expression of the canonical MC marker genes *FCER1A* (High affinity Fc IgE receptor), *CD48*, *FCER1G* (Fc IgE receptor), *MS4A2* (IgE subunit) and *ITGAX* (CD11c) as a negative marker. The cells identified MC cluster (only one cluster) were subset and re-clustered using the method outlined above at a resolution of 0.1. To identify marker genes for mast cells, we used *FindAllMarkers* to compare cluster against all other clusters, and *FindMarkers* to compare selected clusters. For each cluster, only genes that were expressed in more than 15% of cells with at least 0.15-fold differences were considered. The differential genes were subjected to enrichment analysis using Hallmark Pathway gene set from *MsigDB*. The pathways that met an FDR threshold less than 0.05 were considered.

Gene signatures were defined with R package *Ucell*. The output is a module signature score generated by *AddModuleScore* function. The obtained score was overlaid on the UMAP and visualized. The Values per cell were extracted and used to plot a summed module U cell score. GraphPad prism was used for the Violin plots and the heatmap. All other figures were generated in R.

## Results

### Mast cells localize and transition phenotypes within TB granulomas

**Figure 1.**
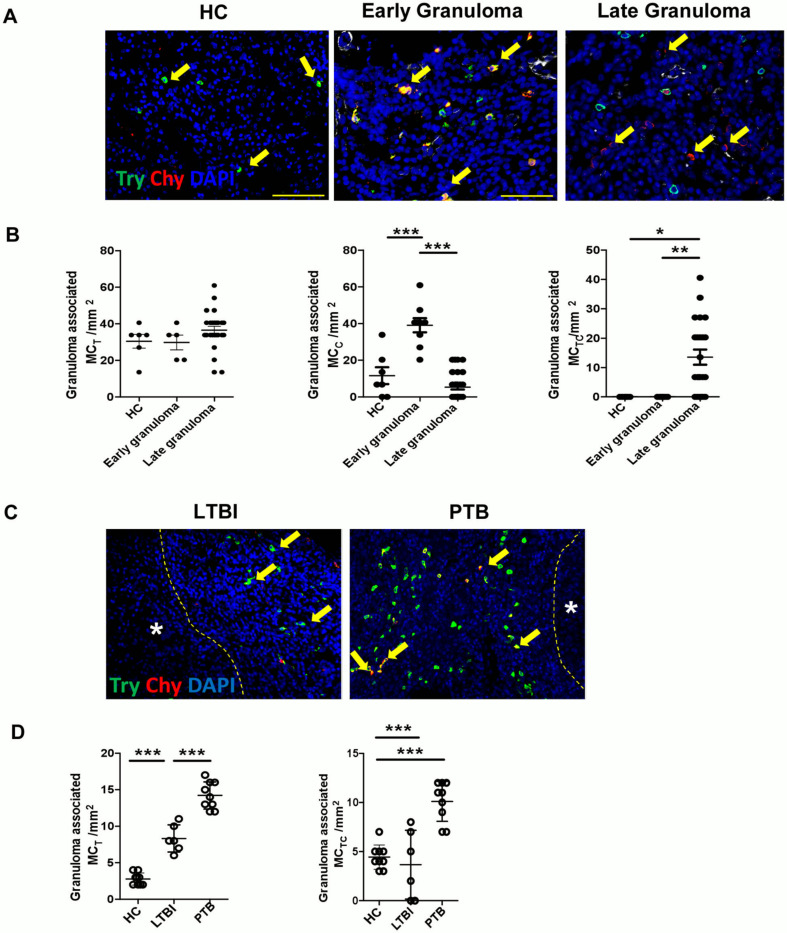
Chymase positive mast cells are predominant in TB infected human and macaque lung tissue. Lung biopsies from healthy individuals (n = 4) or patients with PTB (n = 5) were stained for tryptase MC_T_ (green) or chymase MC_C_ (red). (A) Immunofluorescence microscopy shows MC_TS_ (green) in healthy lung biopsies (HC). MC_TCS_ (red and green merge) are located around the early granulomas, while MC_CS_ (red) surround the late granulomas in TB infected lung biopsies. (B) Predominance of MC_TS_ in healthy lungs transitioning to MC_TCS_ in early granuloma and becoming MC_CS_ in late granulomas in TB infected lungs. (C) Immunofluorescence microscopy shows MC_TS_ (green) and MC_TCS_ (merge) in lungs of healthy (HC), LTBI and PTB macaques. (D) Predominance of MC_TS_ (green) and MC_TCS_ (merge) in PTB compared to LTBI and HC. Statistical analysis was performed using unpaired, 2-tailed Student’s t test, **** p < 0.0001, *** p < 0.001, * p< 0.05.

MC_T_s were primarily found within the lung from healthy controls (HC), while MC_C_s or MC_TC_s were found in the lung biopsies of patients with PTB (Garcia-Rodriguez et al., 2021). To validate these observations and analyze the compartmentalization of MCs in human lungs, we stained lung biopsies from healthy individuals and patients with PTB to visualize the spatial distribution of MC_C_s, MC_T_s and MC_TC_s. Our results show that MC_C_s are not well represented in the lung parenchyma, interstitium, vasculature, or bronchus of HC lungs (Figure 1A, Figure S1). Instead, we observed that the healthy lungs predominantly contain MC_T_s and, to a lesser extent, MC_TC_s. While MC_TC_s accumulated in early immature granulomas within TB lesions, MC_C_s accumulated in late granulomas in TB patients (Figure 1A and B). MC_T_s also increased in the interstitium, vasculature and bronchus-associated lymphoid tissue of patients with PTB (Figure S1A). Therefore, our study confirmed that MC_T_s are found in HCs (Garcia-Rodriguez et al., 2021) and likely convert first to MC_TC_s in early granulomas before becoming MC_C_s in late mature granulomas with necrotic cores.

Our published data showed that MCs accumulate in the lungs of macaques with PTB compared to LTBI macaque lungs (Esaulova et al., 2021). Thus, we next analyzed the accumulation and localization of MCs in the lungs of macaques with LTBI and PTB. We found that similar to human healthy lungs, MC_T_s accumulated in the lungs of healthy macaques. Although MC_T_s and these cells increased in some lesions in the lungs of macaques with LTBI, the number of MC_T_s in macaques with PTB were significantly increased in all sites including granuloma (Figure 1C and D), interstitium as well as vasculature (Figure S1B). Additionally, while MC_TC_s increased in the granulomas of macaques with PTB compared to the lungs of macaques with LTBI and HCs, we did not observe any increase of these cells in other sites within the lung compared to healthy macaques. No MC_C_s were measurable within macaque lungs. Our data indicate an accumulation of MC_T_s but not MC_TC_s during LTBI. However, as the disease progresses to PTB, there is an increase in both MC_TC_s and additional MC_T_s.

### Lung mast cells express inflammatory and metabolically active transcriptome in macaques with PTB

**Figure 2.**
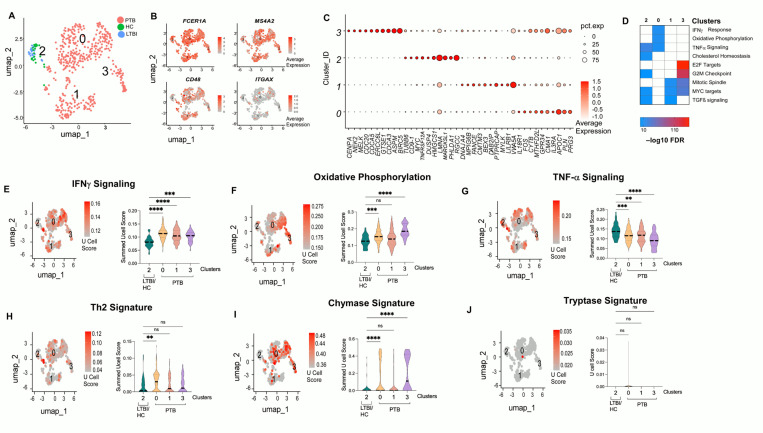
Mast cells in lung in active TB show an inflammatory and metabolically active transcriptome as compared to HC or LTBI. Single-cell (sc) RNA-seq Data was re-analyzed from the lung of non-human primates (Esauolva *et al*). (A) UMAP embedding of the *FCER1A*^+^ mast cells, revealed four transcriptionally distinct clusters; the distribution of the MCs across disease condition is indicated by the color PTB (pink), HC (green) and LTBI (blue). (B) Showing the average expression of MCs specific marker genes (*FCER1A*, *MS4A2* and *CD48*) and a macrophage gene *ITGAX* in contrast. (C) Dot plot indicating expression of top differentially expressed genes detected for each cell cluster identified. The dot color represents the expression level, and the dot size represents the percentage of cells in each cluster expressing a particular gene. (D) Hallmark Pathway analysis of the differential genes, only top pathways with highest FDR in each cluster is plotted. The color indicates the –log10 FDR; E-H) UMAP plots with the U cell module score (averaged score of all genes) of the pathways and their corresponding violin plots. Cluster 2 from LTBI/HC were compared to the rest of the PTB groups using a Kruskal-Wallis test with Dunn’s multiple correction. **** p < 0.0001, *** p < 0.001, ns: not significant.

To further understand the transcriptional differences between MCs in lungs of HCs, LTBI and PTB, we re-analyzed the MC subpopulation in the scRNA seq data from macaques from Esauolva et al. We examined the single-cell transcriptome of 500 mast cells with unsupervised clustering and identified four clusters, three of which (0,1,3) belonged to PTB group and cluster 2 was found exclusively in LTBI and HC (Figure 2A). All the MC clusters were positive for markers such as *FCER1A* (high-affinity IgE receptor), *MS4A2* (IgE subunit), *CD48* (mast cell receptor) and negative for markers like *ITGAX* (macrophage/dendritic cell marker) (Figure 2B). Differential expressed genes (DEGs) among the cluster revealed cluster specific markers. Among the top 10 genes of the largest PTB cluster 0 were *CENPA* (metabolic reprogramming related centromeric protein, (Liang et al., 2021)), *NEK2*, *MELK2* (NF-kB regulator), (Zhang et al., 2022)), *CDC20* (role in LC3 mediated autophagy; (Xie et al., 2018)), *CDCA5*, *GTSE1*, *CDCA3, ASPM* (proliferation related, (Shen et al., 2021), ASPM; (Kouprina et al., 2005)), and *BIRC5* (Immunosuppressive and infiltration-associated), genes that were upregulated in MCs from PTB compared to MCs from LTBI and HC lungs. Similarly, the transcriptome of the cells from LTBI and HC expressed high levels of *TUBB6* (inhibitor of pyroptosis, (Salinas et al., 2014)), *CD9–1*, *TNFRSF12A* (TNF receptor, needed for anti-TB immunity), *DUSP4, HMGCS1*, *LMNA*, *MARCKSL1*, *PHLDA1*, *RGCC* as compared to PTB clusters. The other PTB clusters expressed genes such as *FANCE*, *IL18R1*, *FOS*, *CYTB*, *IL3RA* are genes related to activated mast cells (Figure 2C). Enrichment of cluster specific DEGs revealed enrichment of cholesterol, TNF-α, and TGFβ signaling in LTBI. Oxidative phosphorylation, and IFNγ signaling, and MYC signaling were enriched in the PTB group (Figure 2D). Plotting the summed *Ucell* module scores revealed significant upregulation of IFNγ signaling, Oxidative Phosphorylation and Th2 signature in PTB (P < 0.05), while LTBI and HC clusters showed enhanced TNF signaling (P <0.05) (Figure 2E-H). Since we observed increased chymase in MCs of PTB (Figure 1A), we used a chymase signature to compare the PTB with LTBI / HC mast cells. The MCs from PTB (Cluster 0) expressed high levels of chymase, confirming the immunostaining results (Figure 1A). Thus, this analysis provides evidence for the transcriptional heterogeneity in the MCs derived from PTB and LTBI and shows differential activation programs in the cell based on the disease state.

### MC deficient mice exhibit enhanced control of *Mtb*

We next determined if MCs are induced in response to infection in mice following aerosolized low dose *Mtb* infection, and whether they accumulate early or later in infection. Our prior results have shown that innate cells such as ILCs accumulate very early between days 5–10 and followed by accumulation of other innate cells such as neutrophils, macrophages and monocytes between days 10–15 and T cells by day 21 to 30 (Ardain et al., 2019). We found that MCs accumulate in the lung at time points between day 21 and 30 and coincide with timing of accumulation of T cells in the lung (data not shown).

**Figure 3:**
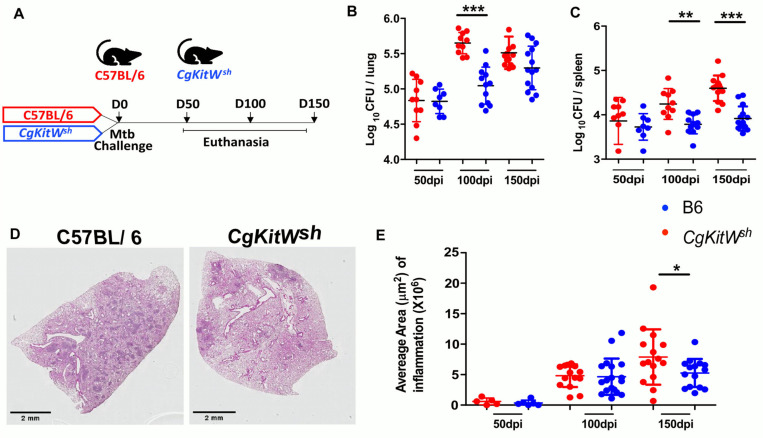
MC deficient mice are resistant to *Mtb* chronic infection. (A) C57BL/6 and CgKitW^sh^ mice were infected with a low aerosol dose (~100CFU) of *Mtb* HN878 and mice were sacrificed at 50, 100 and 150 dpi. (B) Bacterial burden was assessed in lungs and spleens by plating. (C) Lungs were harvested, fixed in formalin and embedded in paraffin. H&E staining was caried out for blinded and unbiased analysis of histopathology. (D) Representative images and the area of inflammation measured in each lobe is shown. Scale bars: 2mm. Original magnification: ×20. Data points represent the mean ± SD of two experiments (*n* = 8–15 per time point per group). Statistical analysis was performed using unpaired, 2-tailed Student’s t test between C57BL/6 and *CgKitW*^*sh*^ mice, **** p < 0.0001, *** p < 0.001, * p< 0.05.

To further investigate the functional role of MCs in *Mtb* infection, we utilized MC deficient mouse model, *CgKitW*^*sh*^. Mice carrying spontaneous loss-of-function mutations at both alleles of the dominant *white spotting* (*W*) locus (*i.e.*, c-*kit*) exhibit a marked reduction in c-*kit* tyrosine kinase-dependent signaling resulting in dysregulated mast cell development, survival and function (Wolters et al., 2005). We infected *CgKitW*^*sh*^ mice with low dose aerosolized *Mtb* HN878 and found that compared with wild type control *Mtb*-infected mice, *CgKitW*^*sh*^ mice did not show any differences in *Mtb* CFU at early time points (50 days post infection (dpi), (Figure 3A). However, at later time points (100 dpi and 150 dpi), *CgKitW*^*sh*^ *Mtb*-infected mice showed significantly lower lung *Mtb* CFU, when compared with *Mtb*-infected wild type control mice (Figure 3B). This coincided with reduced inflammation in the lungs of *CgKitW*^*sh*^ *Mtb*-infected mice at 150 dpi (Figure 3D and E). Additionally, no differences in *Mtb* CFU were observed at 50 dpi in the spleen, *CgKitW*^*sh*^ *Mtb*-infected mice showed enhanced control of *Mtb* in the spleen at 100 and 150 dpi (Figure 3C). Therefore, *CgKitW*^*sh*^ mice lacking MCs exhibit better *Mtb* control, specifically during late stages of infection.

**Figure 4:**
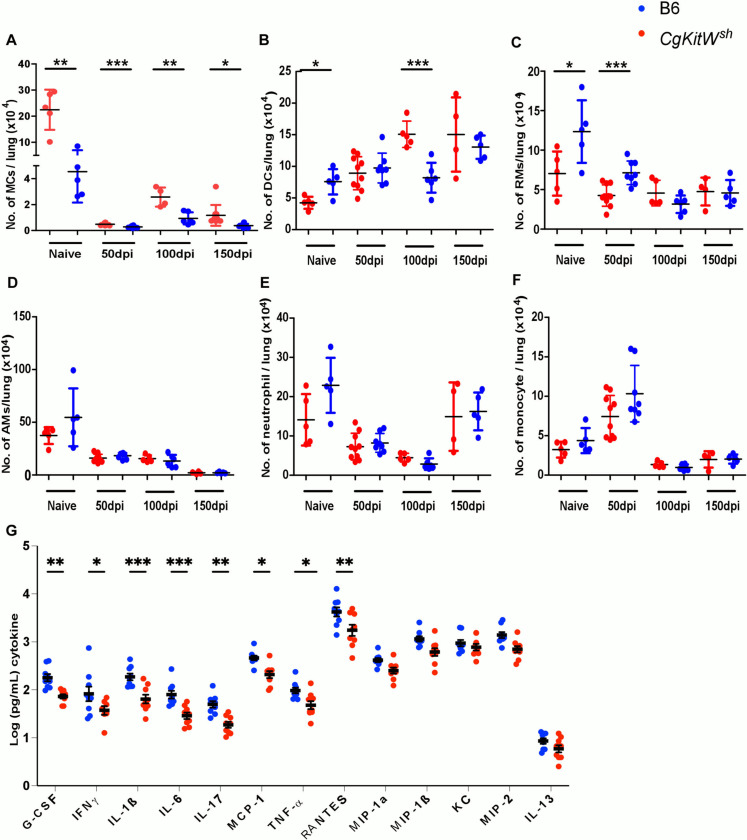
MC deficient mice have dysregulated immune profile after *Mtb* infection. C57BL/6 and *CgKitW*^*sh*^ mice were infected with a low aerosol dose (~100CFU) of *Mtb* HN878 and mice were sacrificed at 50, 100 and 150 dpi. Number of (A) mast cells, (B) dendritic cells, (C) recruited macrophages, (D) alveolar macrophages, (E) neutrophils and (F) monocytes were enumerated in the lungs of *Mtb* infected mice. (G) Cytokine and chemokines production in lung homogenates from mice, collected at 150dpi, was assessed by multiplex cytokine analysis. Data points represent the mean ± SD of 1 of 2 individual experiments (*n* = 5–8 per time point per group). Statistical analysis was performed using unpaired, 2-tailed Student’s t test for (A) to (F) and Two-way ANOVA Sidak’s multiple comparison test for (G) between C57BL/6 and *CgKitW*^*sh*^ mice, *** p < 0.0001, ** p < 0.001, * p< 0.05. Outliers were removed from the 616 subsets using Grubb’s outlier test.

To mechanistically address the functional basis of enhanced protection, we analyzed the lungs of *CgKitW*^*sh*^ mice before and after *Mtb* infection as this mouse strain is associated with other known immune deficiencies (Grimbaldeston et al., 2005). At baseline, MCs were significantly reduced in the lungs of *CgKitW*^*sh*^ mice compared to B6 mice. MCs continued to accumulate in the lung up to 100 dpi in *CgKitW*^*sh*^ mice, following which the MC numbers decreased at later stages. Indeed, the deficiency in MCs observed in lungs of *CgKitW*^*sh*^ mice was retained following infection until 150 dpi (Figure 4A). However, we also found that at baseline *CgKitW*^*sh*^ mice also showed increased dendritic cells (DCs) as well as recruited macrophages (RMs) but no perturbations in alveolar macrophages (AMs), neutrophils or monocytes. Additionally, the increased DCs and RMs responses were also evident in *CgKitW*^*sh*^ mice at 50 dpi following infection, but not maintained at later time points (Figure 4B and C). No differences in AMs, neutrophils and monocytes were observed in the lungs of *Mtb*-infected *CgKitW*^*sh*^ mice when compared with *Mtb*-infected control mice at all time points tested (Figure D, E and F). Previous studies have implicated MCs in driving T cell responses (Elieh et al., 2018). At baseline, we found that there is an increase in CD4^+^ and CD8^+^ T cells numbers (data not shown), we found no differences in CD4^+^ and CD8^+^ T cells responses at 50 dpi, but numbers of activated CD4^+^ and CD8^+^ T cells were significantly reduced at 100dpi, which compromised CD4^+^ IFNγ producing cells as well as TNF-α and IFNγ dual cytokine producing subsets at 100 dpi, in the *CgKitW*^*sh*^ mice as compared to C57Bl/6 *Mtb*-infected mice (Figure S2). Finally, we measured cytokine responses in the lungs of wild type C57Bl/6 and *CgKitW*^*sh*^ *Mtb*-infected mice at 150 dpi. We found that while proinflammatory cytokines that direct monocyte/macrophage responses and T cell responses including G-CSF, IFNγ, IL-1β, IL-6, IL-17, MCP-1, TNF-α and RANTES were significantly higher in wild type *Mtb*-infected lungs compared with *CgKitW*^*sh*^ *Mtb* infected lungs, chemokines that direct neutrophil responses such as MIP-1α, MIP-1β, KC and MIP2 and Th2 cytokines like IL-13 were not different between wild type and *CgKitW*^*sh*^ *Mtb*-infected lungs at 150 dpi (Figure 4G). These results together provide evidence that MCs are induced following *Mtb* infection, accumulate in the lung and mediate cytokine responses to drive pathology and promote *Mtb* susceptibility and dissemination during TB.

## Discussion

The immune mechanism(s) that mediate the progression from LTBI to PTB are unclear. In this study, we identified MCs as an innate cell type that is overrepresented during PTB, transcriptionally express signatures associated with IFNγ, oxidative phosphorylation, and MYC signaling, and localize within mature TB granulomas. Importantly, using mice deficient in MCs, we show a potential pathological role for MCs in mediating susceptibility to TB. Together, this paper provides evidence of MCs in the context of pathology and susceptibility to TB providing MCs as a novel therapeutic platform.

MCs have been shown to interact with *Mtb* through the GPI anchored molecule CD48 (Munoz et al., 2003), interaction with TLR2 (Carlos et al., 2007) and potentially TLR4 (McCurdy, Lin, & Marshall, 2001). Additionally, *Mtb* is also thought to be internalized by lipid rafts on MCs (Munoz et al., 2009), thus serving as a long lasting reservoir for *Mtb* (da Silva et al., 2014). In in vitro studies, MC exposure to *Mtb* resulted in degranulation of MCs as well as induction of proinflammatory cytokines such as TNF-α and IL-1β. While these studies using high dose model of infection reported early induction of inflammatory mediators from MC within hours to days, our in vivo results using a physiological low dose of *Mtb* infection model show that MCs accumulate between 21–30 days coinciding with accumulation of T cells in the lung. Interestingly, despite accumulation of MCs in the lung at 30 dpi following low dose aerosol infection, the impact of MC deficiency on *Mtb* control and inflammation in *CgKitW*^*sh*^ mice is not evident until 100 dpi. This is similar to our published studies where we found that S100A8/9 deficiency resulted in reduced neutrophil lung accumulation (Scott et al., 2020), resulted in improved *Mtb* control and improved TB disease, but after 100 dpi. This is in contrast to the role of eosinophils in TB, where eosinophil deficiency resulted in increased *Mtb* CFU (Bohrer et al., 2021). MCs are the primary cell type defective in the lungs of *CgKitW*^*sh*^ mice at baseline and at all time points following infection, thus, we attribute the improved *Mtb* control with MC deficiency. However, we do report increased RMs and DCs accumulation at baseline in *CgKitW*^*sh*^ mice, but these changes are not maintained until 100 dpi, a time point when we see impact on *Mtb* control. Additionally, the reduced inflammation observed at 100 and 150 dpi is associated with decreased level of proinflammatory cytokines and chemokines that recruit monocytes/macrophages and T cells, but not chemokines that are associated with neutrophil recruitment. These results imply an important role for MCs in amplifying macrophage/monocyte accumulation during chronic TB and driving increased lung pathology. Overall, based on our results along with the current literature, we propose pathological roles for neutrophils and MCs, while other granulocytes such as eosinophils may mediate protective roles (Bohrer et al., 2021).

MCs can release cytokines and chemokines, antimicrobial peptides and granules upon pathogen sensing and to control pathogens (Naqvi et al., 2020). In the context of *Mtb* exposure, MCs have been shown to undergo degranulation including histamine and β-hexosaminidase (Munoz et al., 2003). Indeed, histamine deficient mice showed decreased neutrophils, as well as proinflammatory cytokine production following *Mtb* infection (Carlos et al., 2009). Additionally, induction of degranulation following intratracheal *Mtb* infection resulted in reduced proinflammatory cytokines as well as reduced lung inflammation. With respect to *CgKitW*^*sh*^ *Mtb*-infected mice, except lower numbers of DCs and CD4^+^ and CD8^+^ T cell responses at 100 dpi, we did not find any significant differences in accumulation of innate or adaptive myeloid populations in the lung at other time points. Incidentally decreased numbers of DCs and T cells at 100 dpi was reflected with reduced lung inflammation at 100 dpi. Thus, together with published work, while MCs can potentially modulate neutrophil and other inflammatory mediators in high dose models, in a physiologically relevant *Mtb* infection model, absence of MCs impacted late TB disease and improved *Mtb* control without significant changes to other innate cells. The exact mechanism by which MCs contributing to pathology, dissemination and promoting *Mtb* is an area of future investigation.

At baseline, human lungs have been reported to primarily express tryptase (Poto et al., 2022) Indeed, we found that this is true for both macaque and human lungs in our study where healthy lungs expressed MC_T_s. Additionally, we found that early granulomas and in LTBI we saw expression of MC_TC_s with a switch to more and accumulation of MC_C_s in late stage granulomas. Chymase expression may modulate extracellular matrix components (ECM) such as fibronectin leading to tissue remodeling, impacting cellular communication and inducing cleavage for key cytokine such as IL-6, IL-13, IL-15, and IL-33, as well as Transforming Growth Factor (TGF-β) (Pejler, 2020; Waern et al., 2013). Studies have shown that tryptase can induce proliferation of fibroblasts, epithelial cells and smooth muscle cells, causing airway remodeling during diseased conditions (Mogren et al., 2021). Tryptase can also inactivate a large range of peptides by cleaving specific substrates, such as fibrinogen, gelatin, pro-matrix metalloproteinases (MMP) and complement factors thus moderating inflammatory responses (Caughey, 2007). Based on our results from human and macaque lung, we hypothesize that MC_TC_s may synergize to drive responses induced by both pathways at early time points and possibly just by MC_C_s at later time points. Additional studies describing these subsets and testing their functional relevance in *in vivo* models are future steps in delineating the role of these subsets in TB.

Analysis of the single cell transcriptome from lungs of macaques identified that the MCs from PTB animals show closer resemblance to MC_CS_ with higher IFNγ, metabolic activation and expression of *CMTA* gene and chymase signature, although all the MCs (from PTB, LTBI and HC) had a minimal tryptase signature. MCs are known to gear towards a Th2 signature with increased chymase expression (Toru et al., 1998). This increase was reflected in MCs from the macaque lung, showing a high transcriptomic Th2 signature in PTB but not in clusters found in LTBI and HC. In essence transcriptomics reflected the hyperactivated nature of the MCs in PTB, which might make them more pathologic during infection. Although our *in vivo* mouse study shows pathological role of MCs, significant secretion of Th2 cytokines are not seen, likely because the C57Bl/6 mouse model is prone towards Th1 polarization (Wakeham, et al., 2000). MCs have the ability to release both preformed and de novo synthesized TNF-α, hence helping in early bacterial clearance (Gordon and Galli, 1991). Similarly, our study shows that the MCs from HC and LTBI individuals expressed higher levels of TNF-α, and in less-metabolically activated state (lower OXPHOS signature). So additional studies will help in elucidating the mechanism through which MCs mediate pathological roles.

In summary, we demonstrate that accumulation of chymase producing MC in PTB is a cross-species phenomenon that contributes to increased TB pathology and loss of TB control, thereby elucidating the pathological role of MCs in the control of *Mtb* infection. By targeting MC pathways or signaling mechanisms, host-directed therapies (HDTs) hold the promise of enhancing the effectiveness of existing treatments and mitigating disease-related complications.

## Supplementary Material

Supplement 1

## References

[R1] Ardain A, Domingo-Gonzalez R, Das S, Kazer S W, Howard NC, Singh A, Khader SA. 2019. Group 3 innate lymphoid cells mediate early protective immunity against tuberculosis. Nature 570: 528–532. DOI: 10.1038/s41586-019-1276-2 31168092 PMC6626542

[R2] Bian G, Gu Y, Xu C, Yang W, Pan X, Chen Y, Ma F. 2021. Early development and functional properties of tryptase/chymase double-positive mast cells from human pluripotent stem cells. Journal of Molecular Cell Biology 13: 104–115. DOI: 10.1093/jmcb/mjaa059 33125075 PMC8104937

[R3] Bohrer AC, Castro E, Hu Z, Queiroz ATL, Tocheny CE, Assmann M, Mayer-Barbe KD. 2021. Eosinophils are part of the granulocyte response in tuberculosis and promote host resistance in mice. Journal of Experimental Medicine 218. DOI: 10.1084/jem.20210469 PMC8348215 34347010

[R4] Carlos D, de Souza Junior DA, de Paula L, Jamur MC, Oliver C, Ramos SG, Faccioli LH. 2007. Mast cells modulate pulmonary acute inflammation and host defense in a murine model of tuberculosis. Journal of Infectious Diseases 196: 1361–1368. DOI: 10.1086/521830 17922401

[R5] Carlos D, Fremond C, Samarina A, Vasseur V, Maillet I, Ramos SG, Ryffel B. 2009. Histamine plays an essential regulatory role in lung inflammation and protective immunity in the acute phase of Mycobacterium tuberculosis infection. Infection and Immunity 77: 5359–5368. DOI: 10.1128/IAI.01497-08 19822651 PMC2786437

[R6] Caughey GH. 2007. Mast cell tryptases and chymases in inflammation and host defense. Immunological Reviews 217: 141–154. DOI: 10.1111/j.1600065X.2007.00509.x 17498057 PMC2275918

[R7] da Silva EZ, Jamur MC, Oliver C. 2014. Mast cell function: a new vision of an old cell. Journal of Histochemistry Cytochemistry 62:698–738. DOI: 10.1369/0022155414545334 25062998 PMC4230976

[R8] Esaulova E, Das S, Singh DK, Choreno-Parra JA, Swain A, Arthur L, Khader SA. 2021. The immune landscape in tuberculosis reveals populations linked to disease and latency. Cell Host and Microbe 29: 165–178 e168. DOI: 10.1016/j.chom.2020.11.013 33340449 PMC7878437

[R9] Elieh AKD, Grauwet K. 2018. Role of Mast Cells in Regulation of T Cell Responses in Experimental and Clinical Settings. Clinical Reviews in Allergy and Immunology 54: 432–445.DOI: 10.1007/s12016-017-8646-z 28929455

[R10] Garcia-Rodriguez KM, Bini EI, Gamboa-Dominguez A, Espitia-Pinzon CI, Huerta-Yepez S, Bulfone-Paus S, Hernandez-Pando R. 2021. Differential mast cell numbers and characteristics in human tuberculosis pulmonary lesions. Scientific Reports 11: 10687. DOI: 10.1038/s41598-021-89659-6 34021178 PMC8140073

[R11] Gopal R, Rangel-Moreno J, Slight S, Lin Y, Nawar HF, Fallert Junecko BA, Khader SA. 2013. Interleukin-17-dependent CXCL13 mediates mucosal vaccine-induced immunity against tuberculosis. Mucosal Immunology 6: 972–984. DOI: 10.1038/mi.2012.135 23299616 PMC3732523

[R12] Griffiths K., Ahmed M., Das S., Gopal R., Horne W., Connell TD., Moynihan KD., Kolls JK., Irvine DJ., Artyomov MN., Rangel-Moreno J., Khader SA. 2016. Targeting dendritic cells to accelerate T-cell activation overcomes a bottleneck in tuberculosis vaccine efficacy. Nature Communications 7: 13894. DOI: 10.1038/ncomms13894 PMC5192216 28004802

[R13] Grimbaldeston MA, Chen CC, Piliponsky AM, Tsai M, Tam SY, Galli SJ. 2005. Mast cell-deficient W-sash c-kit mutant Kit W-sh/W-sh mice as a model for investigating mast cell biology in vivo. The American Journal of Pathology 167: 835–848.DOI: 10.1016/S0002-9440(10)62055-X 16127161 PMC1698741

[R14] Gordon J R, Galli S J. 1991. Release of both preformed and newly synthesized tumor necrosis factor alpha (TNF-alpha)/cachectin by mouse mast cells stimulated via the Fc epsilon RI. A mechanism for the sustained action of mast cell-derived TNF-alpha during IgE-dependent biological responses. Journal of Experimental Medicine 174: 103–107. DOI: 10.1084/jem.174.1.103 1829107 PMC2118884

[R15] Khader SA, Bell GK, Pearl JE, Fountain JJ, Rangel-Moreno J, Cilley GE, Cooper AM. 2007. IL-23 and IL-17 in the establishment of protective pulmonary CD4+ T cell responses after vaccination and during Mycobacterium tuberculosis challenge. Nature Immunolog, 8:369–377. DOI: 10.1038/ni1449 17351619

[R16] Kouprina N, Pavlicek A, Collins NK, Nakano M, Noskov VN, Ohzeki J, Larionov V. 2005. The microcephaly ASPM gene is expressed in proliferating tissues and encodes for a mitotic spindle protein. Human Molecular Genetics 14:2155–2165. DOI: 10.1093/hmg/ddi220 15972725

[R17] Liang YC, Su Q, Liu YJ, Xiao H, Yin HZ. 2021. Centromere Protein A (CENPA) Regulates Metabolic Reprogramming in the Colon Cancer Cells by Transcriptionally Activating Karyopherin Subunit Alpha 2 (KPNA2). American Journal of Pathology 191:2117–2132. DOI: 10.1016/j.ajpath.2021.08.010 34508688

[R18] McCurdy JD, Lin TJ, Marshall JS. 2001. Toll-like receptor 4-mediated activation of murine mast cells. Journal of Leukocyte Biology 70: 977–984. 11739561

[R19] Mogren S, Berlin F, Ramu S, Sverrild A, Porsbjerg C, Uller L, Andersson CK. 2021. Mast cell tryptase enhances wound healing by promoting migration in human bronchial epithelial cells. Cell Adhesion and Migration 15, 202–214. DOI: 10.1080/19336918.2021.1950594 34308764 PMC8312598

[R20] Munoz S, Hernandez-Pando R, Abraham SN, Enciso JA. 2003. Mast cell activation by Mycobacterium tuberculosis: mediator release and role of CD48. Journal of Immunology 170:5590–5596. DOI: 10.4049/jimmunol.170.11.5590 12759438

[R21] Munoz S, Rivas-Santiago B, Enciso JA. 2009. Mycobacterium tuberculosis entry into mast cells through cholesterol-rich membrane microdomains. Scandavian Journal of Immunology 70:256–263. DOI: 10.1111/j.1365-3083.2009.02295.x 19703015

[R22] Naqvi N, Srivastava R, Naskar P, Puri N. 2021. Mast cells modulate early responses to Mycobacterium bovis Bacillus Calmette-Guerin by phagocytosis and formation of extracellular traps. Cellular Immunology 365: 104380. DOI: 10.1016/j.cellimm.2021.104380 34049012

[R23] Naqvi N, Srivastava R, Selvapandiyan A, Puri N. 2020. Host Mast Cells in Leishmaniasis: Friend or Foe? Trends of Parasitology 36: 952–956. DOI: 10.1016/j.pt.2020.09.010 33060062

[R24] World Health Organization, 2023. Global Tuberculosis Report Retrieved from https://www.who.int/teams/global-tuberculosis-programme/tb-reports/global-tuberculosis-report-2023.

[R25] Pejler G. 2020. Novel Insight into the in vivo Function of Mast Cell Chymase: Lessons from Knockouts and Inhibitors. Journal of Innate Immunology 12: 357–372. DOI: 10.1159/000506985 PMC7506261 32498069

[R26] Poto R, Criscuolo G, Marone G, Brightling CE, Varricchi G.2022. Human Lung Mast Cells: Therapeutic Implications in Asthma. International Journal of Molecular Sciences 23. DOI: 10.3390/ijms232214466 PMC9693207 36430941

[R27] Salinas RE, Ogohara C, Thomas MI, Shukla KP, Miller SI, Ko DC. 2014. A cellular genome wide association study reveals human variation in microtubule stability and a role in inflammatory cell death. Molecular Biology of the Cell 25:76–86. DOI: 10.1091/mbc.E13-06-0294 24173717 PMC3873895

[R28] Scott NR, Swanson RV, Al-Hammadi N, Domingo-Gonzalez R, Rangel-Moreno J, Kriel BA, Khader SA. 2020. S100A8/A9 regulates CD11b expression and neutrophil recruitment during chronic tuberculosis. Journal of Clinical Investigation, 130: 3098–3112. DOI: 10.1172/JCI130546 32134742 PMC7259997

[R29] Shen D, Fang Y, Zhou F, Deng Z, Qian K, Wang G, Wang X. 2021. The inhibitory effect of silencing CDCA3 on migration and proliferation in bladder urothelial carcinoma. Cancer Cell International 21: 257. DOI: 10.1186/s12935-021-01969-x 33980246 PMC8114508

[R30] Toru H, Eguchi M, Matsumoto R, Yanagida M, Yata J, Nakahata T. 1998. Interleukin-4 promotes the development of tryptase and chymase double-positive human mast cells accompanied by cell maturation. Blood, 91:187–195. 9414284

[R31] Waern I, Lundequist A, Pejler G, Wernersson S. 2013. Mast cell chymase modulates IL-33 levels and controls allergic sensitization in dust-mite induced airway inflammation. Mucosal Immunol 6: 911–920. DOI: 10.1038/mi.2012.129 23235745

[R32] Wakeham J, Wang J, Xing Z. 2000. Genetically determined disparate innate and adaptive cell-mediated immune responses to pulmonary Mycobacterium bovis BCG infection in C57BL/6 and BALB/c mice. Infection and Immunity, 68:6946–6953. DOI: 10.1128/IAI.68.12.6946-6953.2000 11083818 PMC97803

[R33] Wolters PJ, Mallen-St Clair J, Lewis CC, Villalta SA, Baluk P, Erle DJ, Caughey GH. 2005. Tissue-selective mast cell reconstitution and differential lung gene expression in mast cell-deficient Kit(W-sh)/Kit(W-sh) sash mice. Clinical and Experimental Allergy, 35: 82–88. DOI: 10.1111/j.1365-2222.2005.02136.x 15649271 PMC2271075

[R34] Xie YP, Lai S, Lin QY, Xie X., Liao JW, Wang HX, Li HH. 2018. CDC20 regulates cardiac hypertrophy via targeting LC3-dependent autophagy. Theranostics 8: 5995–6007. DOI: 10.7150/thno.27706 30613277 PMC6299438

[R35] Zhang Y, Lu R, Huang X, Yin E, Yang Y, Yi C, Yuan X. 2022. Circular RNA MELK Promotes Chondrocyte Apoptosis and Inhibits Autophagy in Osteoarthritis by Regulating MYD88/NF-kappaB Signaling Axis through MicroRNA-497–5p. Contrast Media Molecular Imaging 2022: 7614497. DOI: 10.1155/2022/7614497 35992546 PMC9356867

