# Supplementary material for: Mast cells promote pathology and susceptibility in tuberculosis
Source: bioRxiv. 2025 Sep 13:2024.09.04.611333. Originally published 2024 Sep 9. Preprint. [Version 2] doi: 10.1101/2024.09.04.611333 (PMC11418949; doi:10.1101/2024.09.04.611333)
Supplement: Supplement 1 [file NIHPP2024.09.04.611333v2-supplement-1.pdf]

bioRxiv preprint doi: https://doi.org/10.1101/2024.09.04.611333; this version posted September 13, 2025. The copyright holder for this preprint (which was not certified by peer review) is the author/funder, who has granted bioRxiv a license to display the preprint in perpetuity. It is made available under aCC-BY 4.0 International license.

Figure S1: Predominance of MC_{TS} in human and NHPs lung interstitium, blood vessels and bronchi. Healthy lung tissue and TB infected biopsies from human and NHP samples were stained for MC_T (green), MC_C (red). Accumulation and localization of (A) MC_{TS}, (B) MC_{CS}, and (C) MC_{TCS} in healthy and TB infected human lung, and (D) MC_{TS} and (E) MC_{TCS} in LTBI and TB macaque lungs. Statistical analysis was performed using unpaired, 2-tailed Student's t test, *** $p < 0.0001$, ** $p < 0.001$, * $p< 0.05$.

bioRxiv preprint doi: https://doi.org/10.1101/2024.09.04.611333; this version posted September 13, 2025. The copyright holder for this preprint (which was not certified by peer review) is the author/funder, who has granted bioRxiv a license to display the preprint in perpetuity. It is made available under aCC-BY 4.0 International license.

Figure S2: Immune cell marker expression and pathway analysis across disease conditions. Data was re-analyzed from the lungs of NHPs (GSE200151). (A) UMAP embeddings of MCs showing expression of cell surface markers: *FCER1A*, *MS4A2*, *CD48*, and *ITGAX* across identified clusters. (B) Dot plot showing the expression levels of select genes across clusters, where the dot size represents the percentage of cells expressing each gene, and the color intensity represents the average expression level. (C) Violin plot of summed module scores for IFNγ signaling, Oxidative Phosphorylation (D), TNF-α signaling (E), Th2 signature (F) pathway across different disease conditions: LTBI/HC (green), PTB (pink), and the respective clusters. (G) UMAP embeddings show the expression of key MC markers, including *TPSG1*, *LOC699599*, and *CMA1* across identified clusters, with color intensity indicating expression levels. Statistical significance was assessed using Kruskal-Wallis tests with Dunn's multiple comparison correction (**$p < 0.01$, ***$p < 0.001$, ****$p < 0.0001$, ns: not significant).

Figure S3: MCs appear at early *Mtb* infection. C57BL/6 mice were infected with a low aerosol dose (~100CFU) of *Mtb* HN878 and mice were sacrificed at 5, 21and 30 dpi. (A) The number of MCs in the lungs was enumerated in *Mtb*-infected mice. (B) Bacterial burden was assessed by plating. Data points represent the mean ± SD (n = 4-5 per time point). Statistical analysis was performed using an unpaired, 2-tailed Student's t test between time points, *** $p < 0.0001$, ** $p < 0.001$, * $p < 0.05$.

Figure S4: MC-deficient mice have no baseline differences in T cell numbers.

Six weeks C57BL/6 mice and $CgKitW^{sh}$ mice were sacrificed to enumerate baseline numbers of (A) $CD4^+$ T cells, and (B) $CD8^+$ T cells. Data points represent the mean ± SD (n = 4-5 per time point).

bioRxiv preprint doi: https://doi.org/10.1101/2024.09.04.611333; this version posted September 13, 2025. The copyright holder for this preprint (which was not certified by peer review) is the author/funder, who has granted bioRxiv a license to display the preprint in perpetuity. It is made available under aCC-BY 4.0 International license.

Figure S5: MC-deficient mice have reduced numbers of activated CD4$^+$ and CD8$^+$ T cells in the lung. C57BL/6 and CgKitW^{sh} mice were infected with a low aerosol dose (~100CFU) of *Mtb* HN878 and mice were sacrificed at 50, 100 and 150 dpi. Number of (A) CD4$^+$ CD44$^+$T cells, (B) CD4$^+$ CD44$^+$ IFNγ$^+$ T cells, (C) CD4$^+$ CD44$^+$TNF-α$^+$ T cells (D) CD4$^+$ CD44$^+$ IFNγ$^+$ TNF-α$^+$ T cells, (E) CD8$^+$ CD44$^+$T cells, (F) CD8$^+$ CD44$^+$ IFNγ$^+$ T cells, (G) CD8$^+$ CD44$^+$ TNF-α$^+$ T cells, and (H) CD8$^+$ CD44$^+$ IFNγ$^+$ TNF-α$^+$ T cells in the lungs of *Mtb* infected mice. Data points represent the mean ± SD of 1 of 2 individual experiments (n = 4-10 per time point per group). Statistical analysis was performed using unpaired, 2-tailed Student's t test between C57BL/6 and CgKitW^{sh} mice, *** $p < 0.0001$, ** $p < 0.001$, * $p < 0.05$. Outliers were removed from the subsets using Grubb's outlier test.

Figure S6: Lung myeloid cell accumulation did not vary in MC-transferred WT *Mtb*-infected mice.

Bone marrow derived in vitro cultured MCs (n=50,000 cells per mouse) were adoptively transferred into the lung airways of WT mice 7 days before infecting with a low aerosol dose (~100CFU) of *Mtb* HN878. MCs were replenished in these mice at 15 dpi, and mice were sacrificed at 30 dpi. Numbers of (A) MCs, (B) neutrophils, and (C) RMs were enumerated in the lungs of *Mtb*-infected mice. Data points represent the mean ± SD of 1 of 2 individual experiments (n = 4 per group).

bioRxiv preprint doi: https://doi.org/10.1101/2024.09.04.611333; this version posted September 13, 2025. The copyright holder for this preprint (which was not certified by peer review) is the author/funder, who has granted bioRxiv a license to display the preprint in perpetuity. It is made available under aCC-BY 4.0 International license.

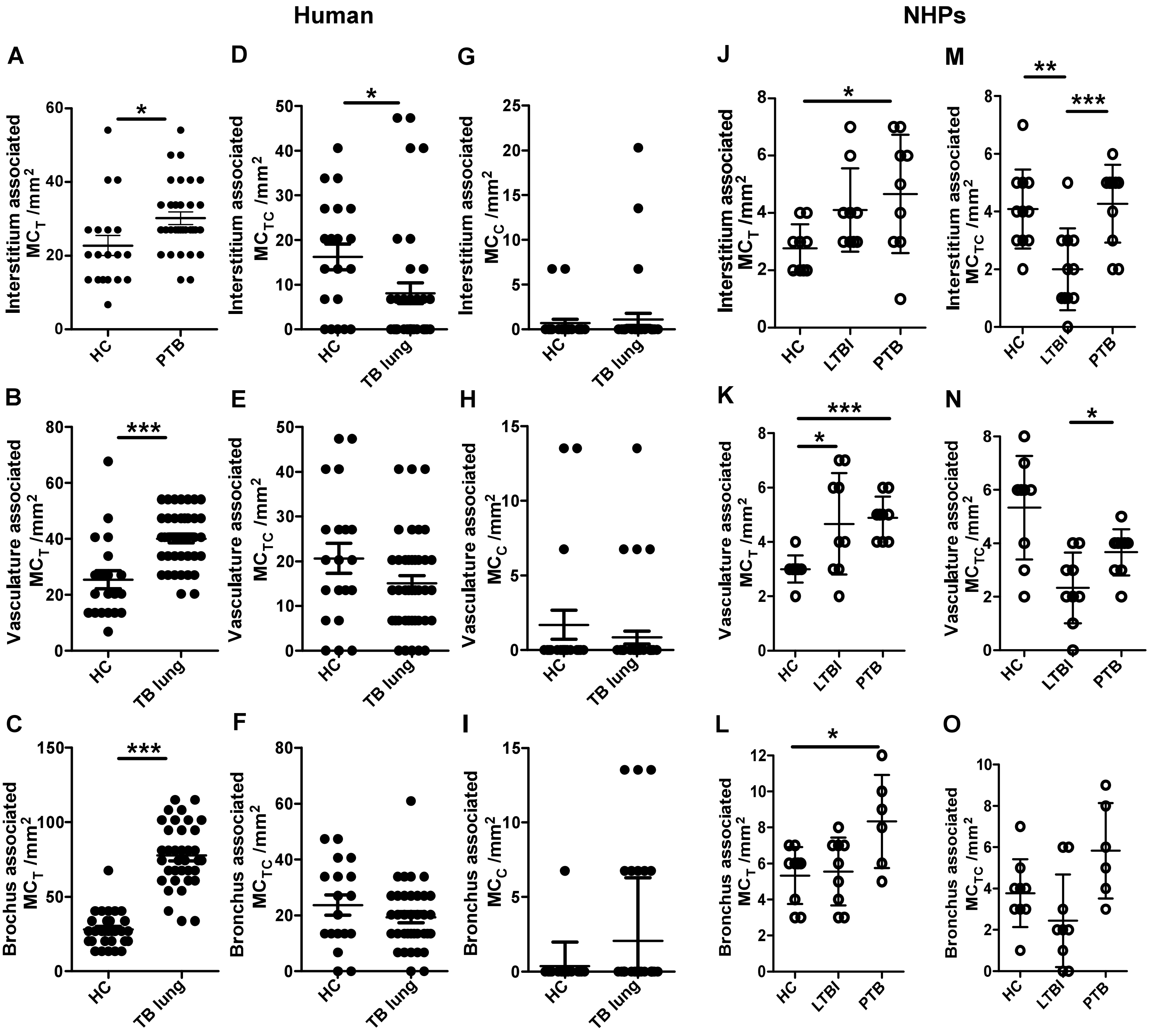

Figure S1

bioRxiv preprint doi: https://doi.org/10.1101/2024.09.04.611333; this version posted September 13, 2025. The copyright holder for this preprint (which was not certified by peer review) is the author/funder, who has granted bioRxiv a license to display the preprint in perpetuity. It is made available under aCC-BY 4.0 International license.

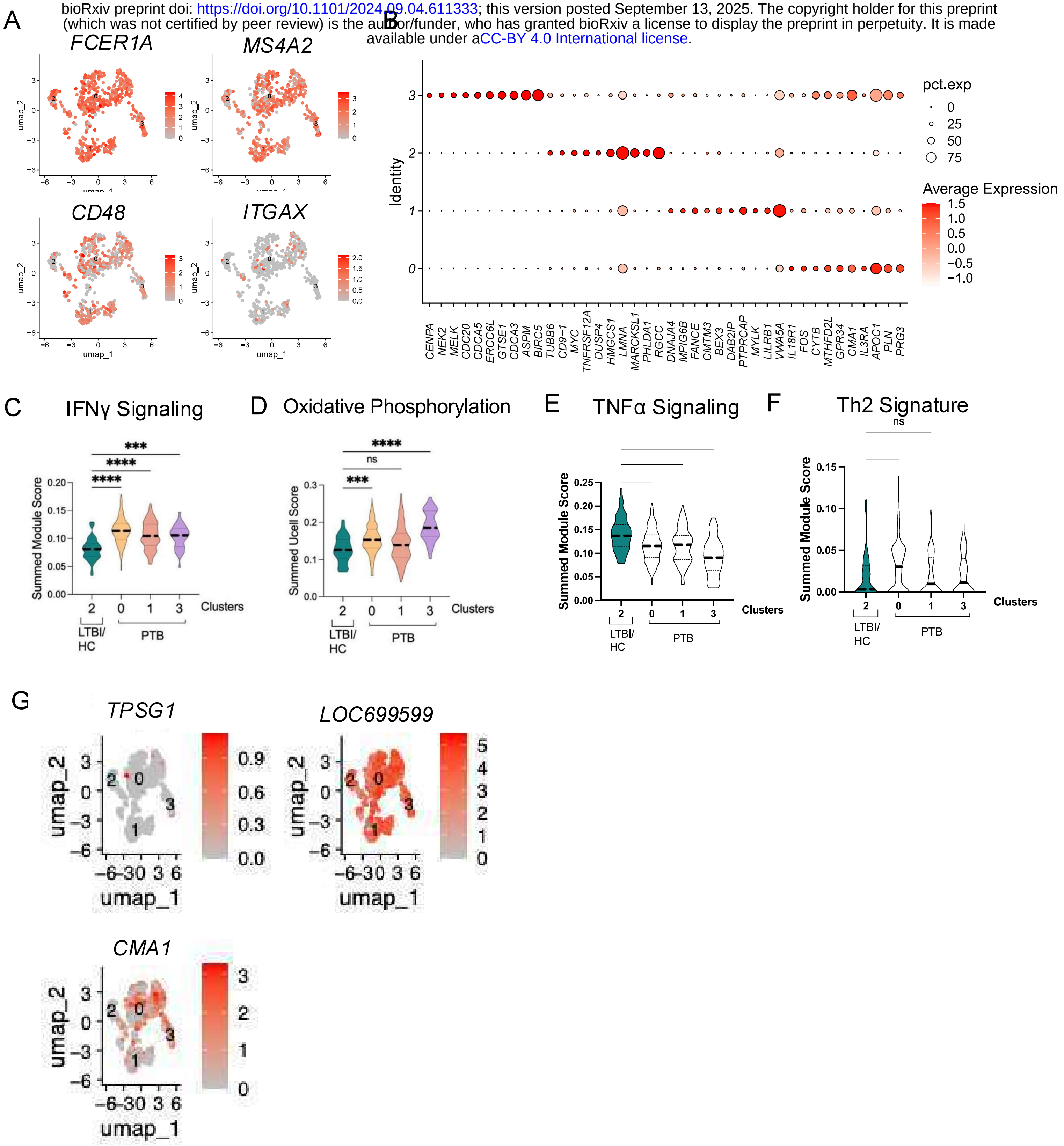

bioRxiv preprint doi: https://doi.org/10.1101/2024.09.04.611333; this version posted September 13, 2025. The copyright holder for this preprint (which was not certified by peer review) is the author/funder, who has granted bioRxiv a license to display the preprint in perpetuity. It is made available under aCC-BY 4.0 International license.

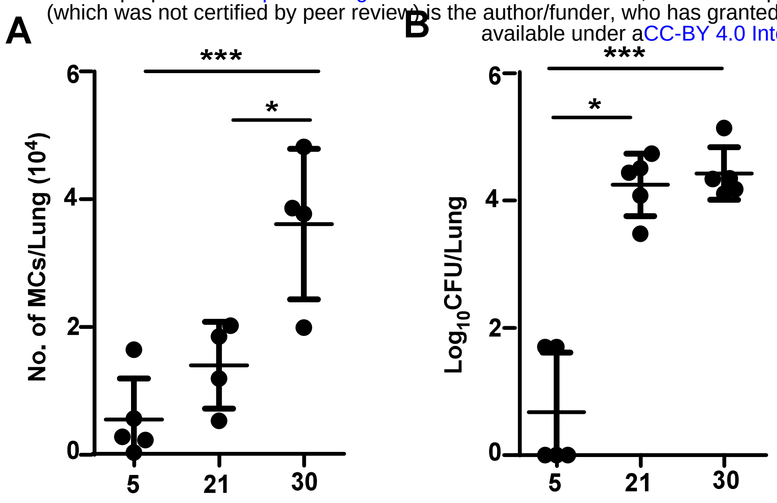

Figure S3

bioRxiv preprint doi: https://doi.org/10.1101/2024.09.04.611333; this version posted September 13, 2025. The copyright holder for this preprint (which was not certified by peer review) is the author/funder, who has granted bioRxiv a license to display the preprint in perpetuity. It is made available under aCC-BY 4.0 International license.

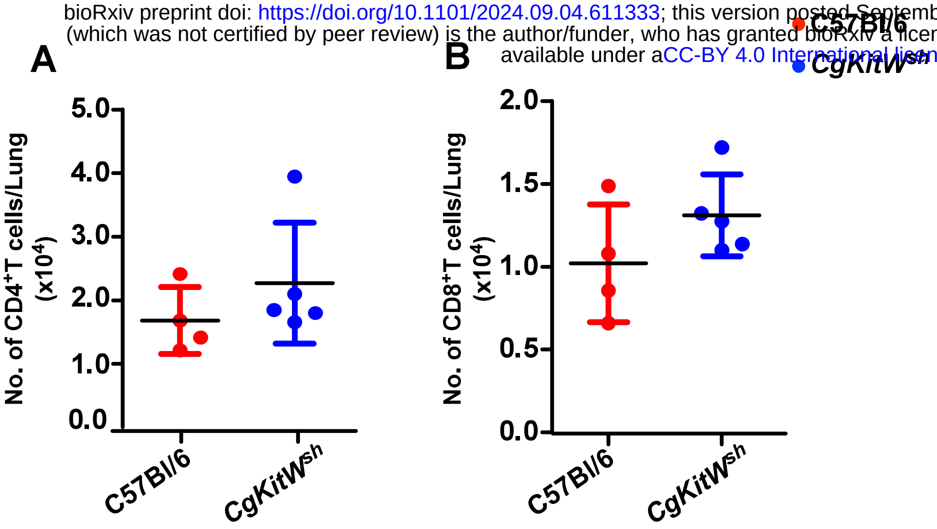

Figure S4

bioRxiv preprint doi: https://doi.org/10.1101/2024.09.04.611333; this version posted September 13, 2025. The copyright holder for this preprint (which was not certified by peer review) is the author/funder, who has granted bioRxiv a license to display the preprint in perpetuity. It is made available under aCC-BY 4.0 International license.

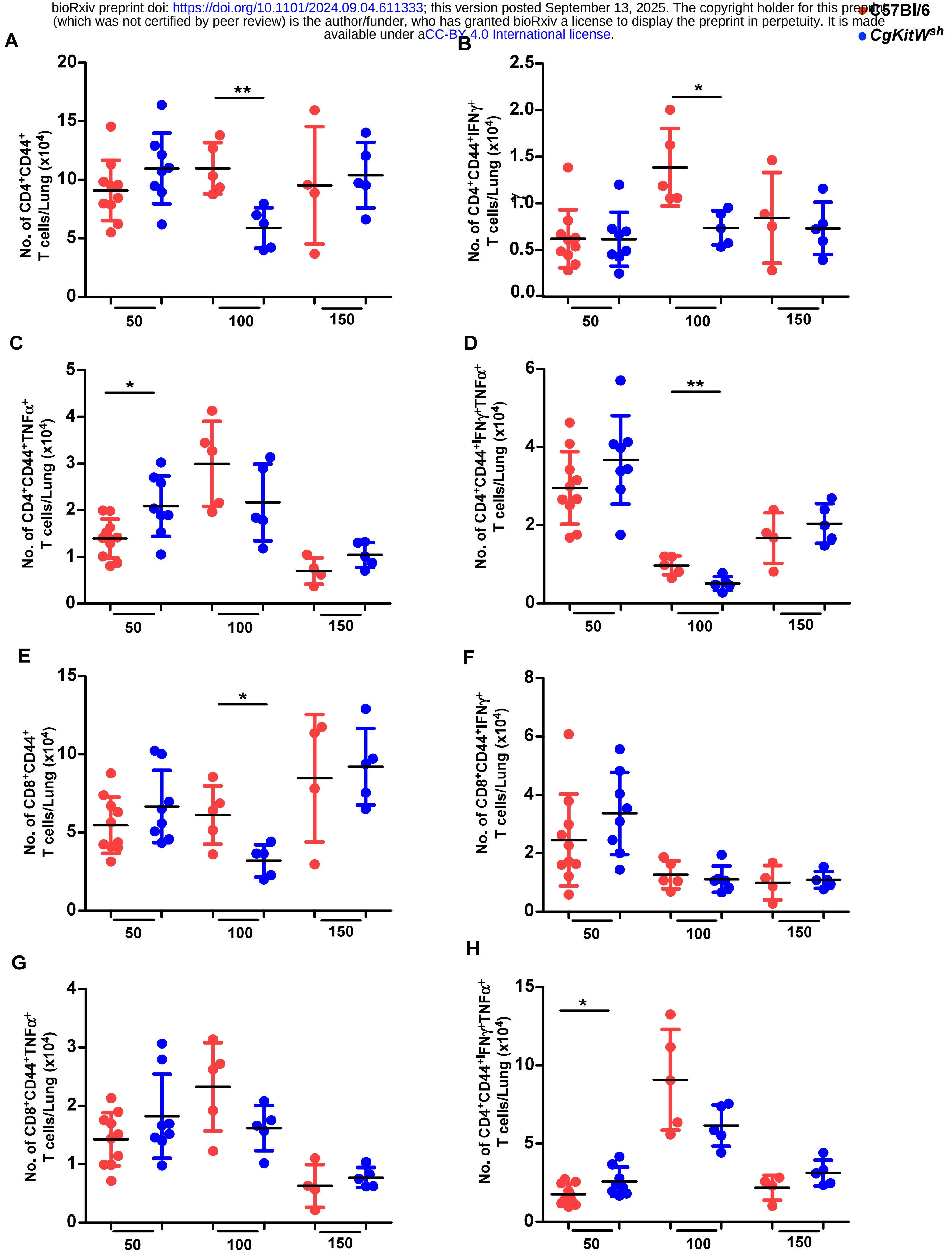

Figure S5

bioRxiv preprint doi: https://doi.org/10.1101/2024.09.04.611333; this version posted September 13, 2025. The copyright holder for this preprint (which was not certified by peer review) is the author/funder, who has granted bioRxiv a license to display the preprint in perpetuity. It is made available under aCC-BY 4.0 International license.

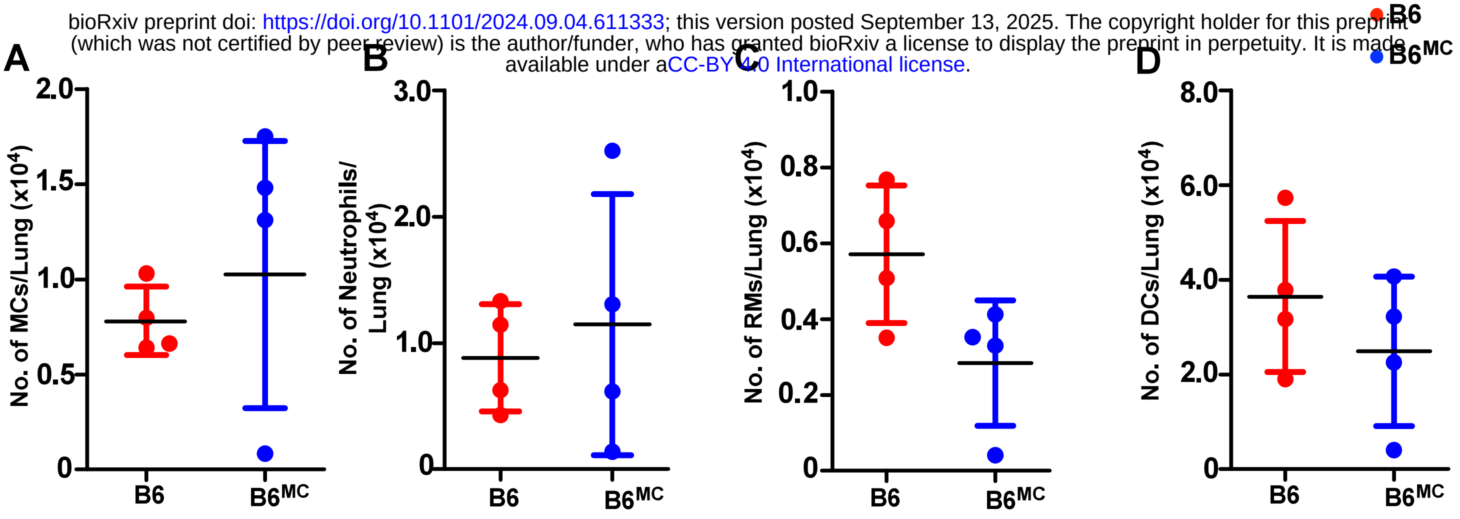

Figure S6
