# Supplementary material for: Mast cells promote pathology and susceptibility in tuberculosis
Source: bioRxiv. 2024 Sep 9:2024.09.04.611333. Preprint. [Version 1] doi: 10.1101/2024.09.04.611333 (PMC11418949; doi:10.1101/2024.09.04.611333)
Supplement: Supplementary file 1 [file NIHPP2024.09.04.611333v1-supplement-1.pdf]

bioRxiv preprint doi: https://doi.org/10.1101/2024.09.04.611333; this version posted September 10, 2024. The copyright holder for this preprint (which was not certified by peer review) is the author/funder, who has granted bioRxiv a license to display the preprint in perpetuity. It is made available under aCC-BY 4.0 International license.

Figure S1: Predominance of MC_{TS} in human and NHPs lung interstitium, blood vessels and bronchi.

Healthy lung tissue and TB infected biopsies from human and NHP samples were stained for MC_T (green), MC_C (red). Accumulation and localization of (A) MC_{TS}, (B) MC_{CS}, and (C) MC_{TCS} in healthy and TB infected human lung, and (D) MC_{TS} and (E) MC_{TCS} in LTBI and TB macaque lungs. Statistical analysis was performed using unpaired, 2-tailed Student's t test, *** $p < 0.0001$, ** $p < 0.001$, * $p < 0.05$.

Figure S2: MC deficient mice have reduced numbers of activated CD4$^+$ and CD8$^+$ T cells in lung. C57BL/6 and CgKitW^{sh} mice were infected with a low aerosol dose (~100CFU) of *Mtb* HN878 and mice were sacrificed at 50, 100 and 150 dpi. Number of (A) CD4$^+$ CD44$^+$T cells, (B) CD4$^+$ CD44$^+$ IFNγ$^+$ T cells, (C) CD4$^+$ CD44$^+$TNF-α$^+$ T cells (D) CD4$^+$ CD44$^+$ IFNγ$^+$ TNF-α$^+$ T cells, (E) CD8$^+$ CD44$^+$T cells, (F) CD8$^+$ CD44$^+$ IFNγ$^+$ T cells, (G) CD8$^+$ CD44$^+$ TNF-α$^+$ T cells, and (H) CD8$^+$ CD44$^+$ IFNγ$^+$ TNF-α$^+$ T cells in the lungs of *Mtb* infected mice. Data points represent the mean ± SD of 1 of 2 individual experiments (n = 5-8 per time point per group). Statistical analysis was performed using unpaired, 2-tailed Student's t test between C57BL/6 and CgKitW^{sh} mice, *** $p < 0.0001$, ** $p < 0.001$, * $p < 0.05$. Outliers were removed from the subsets using Grubb's outlier test.

bioRxiv preprint doi: https://doi.org/10.1101/2024.09.04.611333; this version posted September 10, 2024. The copyright holder for this preprint (which was not certified by peer review) is the author/funder, who has granted bioRxiv a license to display the preprint in perpetuity. It is made available under aCC-BY 4.0 International license.

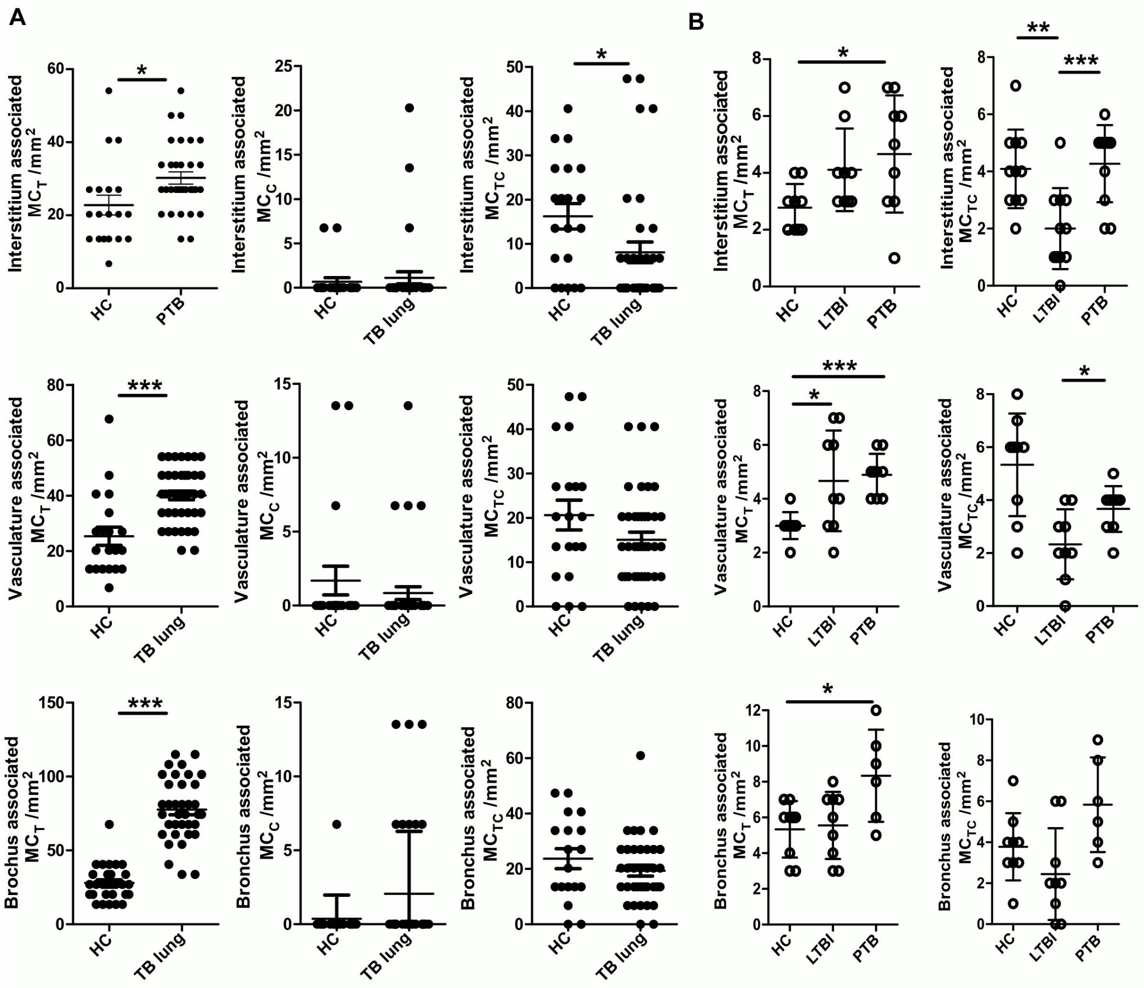

Figure S1

644

bioRxiv preprint doi: https://doi.org/10.1101/2024.09.04.611333; this version posted September 10, 2024. The copyright holder for this preprint (which was not certified by peer review) is the author/funder, who has granted bioRxiv a license to display the preprint in perpetuity. It is made available under aCC-BY 4.0 International license.

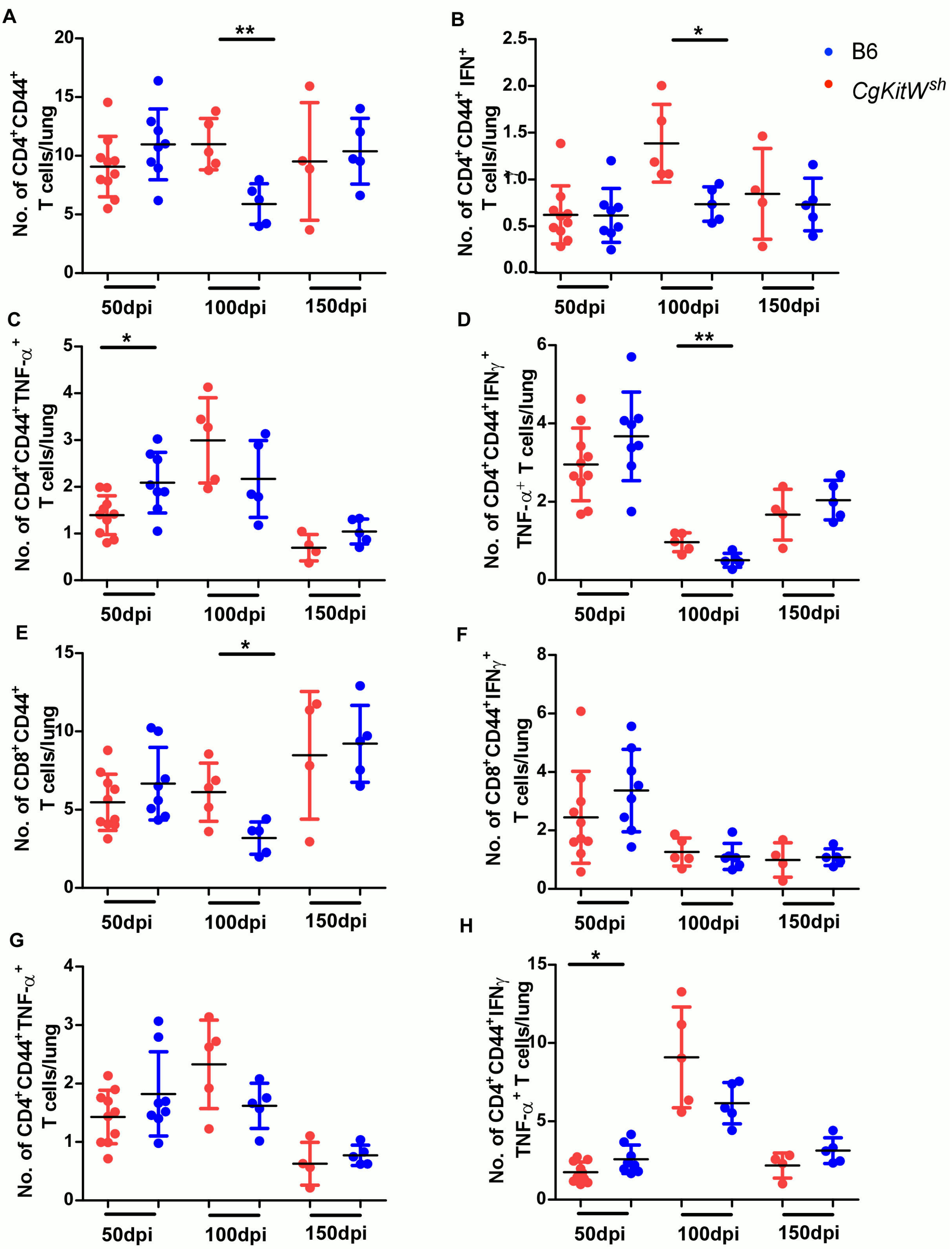

Figure S2

645
